# *Light on Shedding*: A Review of Sex and Menstrual Cycle Differences in the Physiological Effects of Light in Humans

**DOI:** 10.1177/07487304221126785

**Published:** 2022-11-11

**Authors:** Parisa Vidafar, Manuel Spitschan

**Affiliations:** * Turner Institute for Brain and Mental Health, School of Psychological Sciences, Monash University, Melbourne, VIC, Australia; † Translational Sensory and Circadian Neuroscience, Max Planck Institute for Biological Cybernetics, Tübingen, Germany; ‡ TUM Department of Sport and Health Sciences, Technical University of Munich, Munich, Germany; § TUM Institute for Advanced Study, Technical University of Munich, Garching, Germany

**Keywords:** circadian rhythms, light, menstrual cycle, melatonin, biomedical research

## Abstract

The human circadian system responds to light as low as 30 photopic lux. Furthermore, recent evidence shows that there are huge individual differences in light sensitivity, which may help to explain why some people are more susceptible to sleep and circadian disruption than others. The biological mechanisms underlying the differences in light sensitivity remain largely unknown. A key variable of interest in understanding these individual differences in light sensitivity is biological sex. It is possible that in humans, males and females differ in their sensitivity to light, but the evidence is inconclusive. This is in part due to the historic exclusion of women in biomedical research. Hormonal fluctuations across the menstrual cycle in women has often been cited as a confound by researchers. Attitudes, however, are changing with funding and publication agencies advocating for more inclusive research frameworks and mandating that women and minorities participate in scientific research studies. In this article, we distill the existing knowledge regarding the relationship between light and the menstrual cycle. There is some evidence of a relationship between light and the menstrual cycle, but the nature of this relationship seems dependent on the timing of the light source (sunlight, moonlight, and electric light at night). Light sensitivity may be influenced by biological sex and menstrual phase but there might not be any effect at all. To better understand the relationship between light, the circadian system, and the menstrual cycle, future research needs to be designed thoughtfully, conducted rigorously, and reported transparently.

Life on Earth evolved over millions of years on a planet with a strong 24-h light-dark cycle given by solar radiation. Physiology and behavior are subject to endogenous circadian control. The circadian system is the central internal biological clock that keeps to approximately 24 h; regulates the timing of peripheral clocks located in tissues, cells, and organs; and uses light as the primary cue (zeitgeber) to synchronize its daily internal rhythms to the external world (Blume et al., 2019). As diurnal animals, humans evolved to wake up at sunrise, stay active during the day while sunlight was available (e.g., hunt and gather food), and prepare for sleep at sunset. With the advent of electric lighting decoupling light availability from the sun, we can now turn our nights into days with the flick of a switch, extending our work and leisure times well beyond the solar day (Derickson, 2013; Gordon et al., 1986).

The biological connection to the natural light-dark cycle is apparent in studies assessing human sleep and circadian rhythms. Melatonin, a chronobiotic hormone released by the pineal gland, plays a prominent role in the synchronization of the circadian system, and its effects help initiate and maintain sleep in diurnal animals, including humans (Arendt and Skene, 2005; Brainard et al., 1988; Pandi-Perumal et al., 2006; Zeitzer et al., 2000). In healthy individuals, melatonin secretion typically occurs about 2 h before habitual bedtime (Burgess and Fogg, 2008; Burgess et al., 2003; Skocbat et al., 1998), coinciding with the dampening of the circadian signal for alertness (Borbély, 1982). It has a hypothermic effect (lowering core body temperature [CBT]) and peaks when CBT reaches its nadir (typically between 0200 h and 0400 h local time; Brzezinski, 1997; Cajochen et al., 2003; Czeisler, 1978; Czeisler et al., 1980; Duffy and Czeisler, 2009). Exposure to light at night has been shown to suppress melatonin production and secretion, increase sleep onset latency (the time it takes to fall asleep), and delay the timing of sleep (Chang et al., 2015; Santhi et al., 2012; Zeitzer et al., 2000). The suppression of melatonin by light is also dose-dependent, such that the greater the intensity of light, the more melatonin is suppressed (Brainard et al., 1988; Zeitzer et al., 2000).

The scientific understanding of the physiological effects of light on human health and performance outcomes is relatively new. The discovery of a third, non-visual non-image forming physiological photoreceptor class in the human retina just over 20 years ago was ground-breaking (Provencio et al., 1998; Provencio et al., 2000). This non-visual photoreceptor, identified as the intrinsically photoreceptive retinal ganglion cells (abbreviated as ipRGCs), contains a photopigment called melanopsin which is most sensitive to short-wavelength light (Hattar et al., 2002; Lu et al., 2001; Yau et al., 2003; peak absorbance of the pigment ~480 nm, shifted to ~490 nm due to filtering of the lens and ocular media; Spitschan, 2019a). Importantly, the ipRGCs provide a separate signaling pathway from the rods and the cones, of which there are three types with different spectral sensitivities (L, M, and S cones). Accounting for < 5% of all retinal ganglion cells (Freedman et al., 1999), ipRGCs are the primary drivers of the non-visual physiological effects of light (Freedman et al., 1999; Gooley et al., 2010; Hattar et al., 2002; Lu et al., 2001), relaying information about environmental light directly to the suprachiasmatic nuclei (SCN) in the hypothalamus via the retinohypothalamic tract (RHT; Blume et al., 2019). Exposure to short-wavelength light produces the greatest melatonin suppression and alerting effects compared with other wavelengths of light (Brainard et al., 2001; Brainard et al., 1988; Gooley et al., 2010; Lockley et al., 2003; Lockley et al., 2006; Rahman et al., 2014; Revell et al., 2006; Thapan et al., 2001).

Outside of biomedical research, the developing evidence base is currently used in architectural lighting design to create “human-centric lighting” systems in workplaces to increase alertness and productivity (Houser et al., 2021; Houser and Esposito, 2021). Architectural lighting design is big business, with an estimated market size value of $8.1 billion USD in 2020 (Grand View Research, 2020). By 2027, this figure is expected to increase to $12.2 billion USD (Grand View Research, 2020). Various stakeholders collaborate with chronobiologists to establish recommendations and guidelines for lighting systems most beneficial to humans. Given these developments, it is essential to understand how the biomedical evidence base truly reflects human diversity.

A seminal discovery showed that humans are far more sensitive to light at night than previously thought, with light as low as 30 lux (equivalent to a bright smartphone or floor lamp) suppressing melatonin (Zeitzer et al., 2000). To put this in context, it was previously thought that the human circadian system was not sensitive to light at 500 lux (Lewy, 1983; Lewy et al., 1980), with most of the early experiments assessing the effects of light at night in humans using intensities in the range of 200 to 3000 lux (Boyce and Kennaway, 1987; McIntyre et al., 1989; Monteleone et al., 1995; Nathan et al., 1997; Nathan et al., 1999). Of note, experiments predating the standardization of light quantities including the melanopic effects of light generally quantified the biological effect of light using photopic illuminance (in lux), which reflects a combination of the long-wavelength-sensitive (L) and medium-wavelength-sensitive (M) cones in the retina and is distinct from the melanopic effect (CIE, 2018; Schlangen and Price, 2021; Spitschan, 2019c). Chronobiologists are increasingly moving toward measuring the physiological effects of light using the now-standard melanopic equivalent daylight illuminance (m-EDI), which describes the properties of light in relation to the non-image forming ipRGC photoreceptors (Brown, 2020; Lucas et al., 2014).

A recent study evaluated the melatonin-suppressive response to broad spectrum overhead white light ranging from 10 to 2000 lux (5.66-1178.77 m-EDI) in 56 young, healthy males and females of Caucasian descent (Phillips et al., 2019). Each participant was exposed to the various light intensities over 5 to 6 consecutive weeks, and melatonin suppression (used as a marker of light sensitivity) relative to a baseline control (<1 lux, 0.1 m-EDI) was determined in all individuals. At the group level, exposure to 30 lux (17.44 m-EDI) was sufficient to suppress melatonin (Phillips et al., 2019), confirming the earlier finding that the human circadian system is responsive to light as low as 30 lux (Zeitzer et al., 2000). There are huge differences in light sensitivity between individuals, even in a relatively homogeneous sample, which may help explain why some people are more susceptible to sleep, mood, and metabolic disorders than others (Phillips et al., 2019).

Understanding the variables that contribute to these individual differences in light sensitivity is critical for chronobiologists and lighting industries. Without understanding the nuances within and between individuals, the foundation for translating evidence into policy and practice can be less robust. A key variable of interest in understanding these individual differences in light sensitivity is biological sex. It is possible that in humans, males and females differ in their sensitivity to light, but the evidence is inconclusive. In 2020, the human population around the world was reported to be 7.8 billion, 3.8 billion of whom were born with female genitalia. We acknowledge that humans are diverse, and biological sex is not always synonymous with gender identity. Rather, biological sex and gender identity are considered as two separate variables that interact with one another and contribute to an individual’s sense of self. As such, when we use the terms “female/woman/women” and “male/man/men” in this article, we are referring to cisgender women and cisgender men, individuals whose biological sex and gender identity are aligned.

Women have been historically excluded from participating in scientific research studies, with the hormonal fluctuations across the menstrual cycle often cited as a confound. Before the women’s liberation movement of the 1960s and 1970s, most of the experiments that women were involved in pertained to reproductive health and fertility issues (Rich-Edwards et al., 2018; Seaman and Wood, 2000). However, the efforts of women’s advocacy groups led to the National Institutes of Health Revitalization Act in 1993, which mandated the inclusion of women and minorities in scientific research (Rich-Edwards et al., 2018; Seaman and Wood, 2000). These mandates were further reinforced in 2010, and researchers are now required to consider biological sex as a variable in proposed study design, analyses, and reporting (Rich-Edwards et al., 2018). The effect of these mandates is promising, with a recent review showing no routine exclusion of women in clinical research studies carried out in Australia; however, few studies analyzed their results by sex (Rogers and Ballantyne, 2008). In 2009, there were 10 times as many articles examining the menstrual cycle compared with 1974 (Allen et al., 2016).

Although these findings are promising, the historical exclusion of women from participation in biomedical research has undoubtedly left gaping holes in our understanding of sex differences in the response of the circadian system to light at the most basic, mechanistic level. Several studies have assessed the impact of light on the menstrual cycle and neuroendocrine function in women; however, these studies are often limited by small sample sizes, inconsistencies in the determination of menstrual phases, discrepancies in methodologies (Allen et al., 2016; Lee Barron, 2007; Rich-Edwards et al., 2018), and flawed experimental design and analyses (Cutler et al., 1987). The relationship between light and the menstrual cycle is still unclear. It may be the case that women and men differ in their response to light because of biologically different mechanisms. Alternatively, there might not be any differences between the two sexes in their circadian response to light. In any event, if we are to develop recommendations and guidelines for human-centric lighting, we must first understand the basic mechanisms involved in the human circadian system’s response to light. Here, we present an overview of sex and the menstrual cycle in relation to sleep and circadian function, review the few studies that have assessed sex differences in the circadian system’s response to light and the impact of light on the menstrual cycle, discuss the effects of the menstrual cycle on melatonin suppression, and present some strategies for more diversity and inclusivity in future research. The objective of this article is to distill the existing knowledge regarding the relationship between light and the menstrual cycle and encourage effective discourse among scientists in sleep and circadian research.

## Understanding the Menstrual Cycle

The menstrual cycle is a biological phenomenon that is experienced by the majority of women on Earth. The first menstrual cycle, called menarche, typically begins during adolescence (age range 9-16 years; average age ~12 years; Karapanou and Papadimitriou, 2010; Rees, 1995; Zacharias and Wurtman, 1969). The first day of the menstrual cycle starts with menses (menstruation), the shedding of the endometrial layer of the uterus, marked by menstrual bleeding through the vagina (Bull et al., 2019).

The average length of menses (duration of menstrual bleeding) is 3 to 6 days, with 35 to 50 mL of total blood lost during each cycle (total blood loss in excess of 80 mL is considered symptomatic of menstrual disorders; Hallberg et al., 1966). The average length of the menstrual cycle is 28 days, with a normal range of 21 to 35 days (Bull et al., 2019; Chiazze et al., 1968; Vollman, 1977). On average, women will experience about 450 menstrual cycles in their lifetime (Chavez-MacGregor et al., 2008) before transitioning to menopause which can take 4 to 5 years (Joffe et al., 2010; Nelson, 2008). Menopause refers to the reproductive state where ovarian follicles are no longer produced (Gold, 2011) and is determined at 12 months since the last menstrual cycle has occurred (McKinlay et al., 1992; Nelson, 2008). Menopause typically occurs between the ages of 45 to 55 years (Gold, 2011; McKinlay et al., 1972; Nelson, 2008). The function of the menstrual cycle is to prepare the body for pregnancy, and it can be divided into two broad phases: the follicular phase and the luteal phase. Two significant events occur during each of these phases: menstruation (bleeding occurs, signifying the end of the luteal phase and beginning of the follicular phase) and ovulation (egg is released, signifying the end of the follicular phase and beginning of the luteal phase; Allen et al., 2016; Bull et al., 2019).

### The Follicular Phase of the Menstrual Cycle

The follicular phase of the menstrual cycle is characterized by the development of approximately 15 to 20 follicles (Bull et al., 2019; Mihm et al., 2011). During the follicular phase, the hypothalamus promotes the secretion of follicle-stimulating hormone (FSH) from the pituitary gland. FSH signals the release of follicles from the ovaries, with each follicle containing an immature egg (oocyte). Typically, only one follicle will mature into an egg, and the growth of this dominate follicle stimulates the secretion of estradiol (an estrogen steroid and a key sex hormone).

### The Luteal Phase of the Menstrual Cycle

The hypothalamus recognizes the marked increase of estradiol during the end of the follicular phase and responds by releasing gonadotrophin-releasing hormone (GnRH). GnRH stimulates the pituitary gland, which in turn releases a surge of luteinizing hormone (Franco et al., 2014) and FSH (Mihm et al., 2011; Shechter and Boivin, 2010). This short-lived surge of luteinizing hormone (LH) triggers ovulation, where the dominant follicle matures and releases the egg onto the surface of the ovary. Over the next 2 weeks, the follicle transforms into a structure called the corpus luteum. As the corpus luteum develops, it releases progesterone and small amounts of estradiol. The interaction of these sex hormones promotes the thickening of the endometrium. If fertilization takes place within 24 h of the egg being released, human chorionic gonadotrophin is produced (Alonso et al., 2021; Strassmann, 1997). If fertilization does not occur within 24 h, the corpus luteum stops developing, and the decreased levels of progesterone and estradiol cause the shedding of the endometrium (Bull et al., 2019; Strassmann, 1997). Menstruation takes place, and the cycle repeats itself.

### Sex Differences, the Menstrual Cycle, the Circadian System, and Sleep

There is some compelling evidence that sex differences in sleep and circadian parameters exist. Women have earlier bedtimes and wake times, compared with men (Burgard and Ailshire, 2013; Putilov et al., 2008; Reyner and Horne, 1995; Valdez et al., 1996) and generally report more sleep problems such as insomnia and daytime tiredness, as well as a greater need for sleep (Baker et al., 2020; Krishnan and Collop, 2006; Reyner and Horne, 1995; Valdez et al., 1996). Objective evidence from polysomnography recordings show that women have more consolidated sleep than men, have more slow wave activity (a biological marker of sleep drive) than men, and have greater sleep efficiency than men (Putilov et al., 2008; Valdez et al., 1996). These findings suggest that there may be sex differences in the build-up of sleep pressure.

Sex differences in circadian rhythms have also been observed between men and women. Of note, women have an earlier timing of melatonin secretion (Cain et al., 2010) and significantly higher melatonin concentration than men (Cain et al., 2010; Duffy et al., 2011). Women have also been shown to have a shorter intrinsic circadian period (Duffy et al., 2011) compared with men, which is supported by non-human animal research where endogenously applied estradiol shortens the circadian period in rodents (Morin et al., 1977).

The interaction between the menstrual cycle and the circadian system is not fully understood. The infradian (longer than a day) menstrual cycle has a circadian rhythm superimposed upon it as various hormones of the menstrual cycle, such as prolactin and LH, have a daily rhythm (Shechter and Boivin, 2010). Furthermore, estradiol and progesterone receptors have been identified in the human suprachiasmatic nucleus, supporting the notion that these sex hormones directly influence the circadian system (Kruijver and Swaab, 2002). This is an important consideration as the fluctuating hormonal changes across the menstrual cycle may alter sleep and alertness levels in women.

Indeed, results from studies implementing sleep deprivation protocols have shown that there are sex differences in alertness levels between women and men. In one study, reaction times were assessed approximately every 4 h during 40 h of sleep deprivation under highly controlled laboratory conditions. Both younger and older women in this study were shown to have slower reaction times than their male counterparts (Blatter et al., 2006). Women have also been shown to be less accurate on cognitive performance tasks than men under a 28-h forced desynchrony protocol (Santhi et al., 2016). The degree to which these sex differences in alertness can be attributed to hormonal fluctuations across the menstrual cycle has been explored by some researchers.

Evidence from women’s brain health research further suggests a significant effect of female sex hormones on cognitive function. For example, a recent study in healthy, young, naturally cycling (i.e., free from hormonal contraceptives) women found that those with higher salivary estradiol were more worried and had poorer working memory (Gloe et al., 2021). One study showed that women in the follicular phase of their menstrual cycles had lower alertness levels than women in the luteal phase of their menstrual cycles, as well as women using hormonal contraceptives (Wright and Badia, 1999). Another study showed that the sex differences in alertness observed under 30 h of sleep deprivation were primarily driven by the poor performance of women in the follicular phase of their menstrual cycles. Women in the luteal phase of their menstrual cycles, by contrast, performed similarly to, or better than, men (Vidafar et al., 2018).

Together, it appears as though women during the follicular phase of their menstrual cycles show alertness levels that are lower than women in the luteal phase of their menstrual cycles, and subsequently perform worse under sleep deprivation protocols (Vidafar et al., 2018; Wright and Badia, 1999). This poses a potential problem for female night-shift workers, who may feel sleepier on shift during their follicular phase. Recent evidence has shown that when light suppresses melatonin, women in the follicular phase perform similar to women in the luteal phase, suggesting that light exposure may be used as an effective countermeasure for sleepiness on shift (Grant et al., 2020).

On balance, most studies report that slow wave sleep (SWS) remains stable across the menstrual cycle (Driver et al., 1996a; Driver et al., 1996b; Ishizuka et al., 1994; Shechter and Boivin, 2010) while the duration of rapid eye movement (REM) sleep is decreased during the luteal phase (Baker et al., 2002; Driver et al., 1996a; Driver et al., 1996b; Parry et al., 1999; Shechter and Boivin, 2010). It is thought that the decrease in REM sleep is due to the presence of progesterone during the luteal phase. Progesterone has a hyperthermic effect on CBT, raising the temperature by 0.3 to 0.4 °C and attenuating the amplitude of CBT, affecting sleep duration and sleep quality (Baker and Driver, 2006; Kattapong et al., 1995; Shechter and Boivin, 2010; Wright and Badia, 1999). Melatonin production and secretion has generally been shown to remain stable across the menstrual cycle (Allen et al., 2016; Baker and Driver, 2006; Brzezinski et al., 1988; Lee Barron, 2007; Parry et al., 1997; Shechter and Boivin, 2010). There is evidence that light may also have a direct effect on the reproductive organs (Moraes et al., 2021; Yim et al., 2020).

## Does light exposure affect the menstrual cycle?

### The Relationship Between the Lunar Cycle and the Menstrual Cycle

There is a long-standing belief that the lunar cycle and menstrual cycle are somehow connected, with the former influencing the latter. Indeed, the term “menses” itself stems from the Latin word for month (mensis), which is rooted in the Greek word for moon (Tan et al., 2016). There is little scientific evidence at present, however, to support this belief and results are mixed. As early as 1773, French scientist J.A. Murat tracked the menstrual cycle of a woman, believed to be his wife, for 2 years (Lemmer, 2019). In 1806, Murat reported that the menstrual cycle occurs monthly independent from the lunar cycle (Lemmer, 2019). Close to 200 years later, two separate studies claimed there was a significant relationship between the lunar cycle and the menstrual cycle (Cutler et al., 1987; Law, 1986). The data from Law’s (1986) study showed that 28% of women menstruated during the new moon (dark phase), contradicting the findings from Cutler and colleagues (1987) who claimed that most of the women in their study menstruated during the full moon (light phase).

More recently, a year-long retrospective analysis of 980 menstrual cycles in 74 Greek women found no evidence of lunar and menstrual synchronicity (Ilias et al., 2013). Menstrual cycle recordings across six recent cycles were assessed in 529 young Japanese women (aged 25-39 years) and showed no relationship between the onset of lunar and menstrual phases (Komada et al., 2021). These studies analyzed menstrual cycle data in women with varying menstrual cycle lengths, which could explain the null results (Ilias et al., 2013; Komada et al., 2021). In a sizable non-peer-reviewed study, analysis of 7.5 million menstrual cycles collected in 1.5 million users of the period tracker app Clue who were free from hormonal contraceptives, confirmed no association between the lunar cycle and the onset of the menstrual cycle (Science Writers at Clue, 2016). The authors noted that since the lunar phase length is ~29.5 days and the global average of menstrual cycle length is 29 days, one in two women would experience menstruation around the time of the new moon or full moon by chance alone (Science Writers at Clue, 2016).

The most detailed study of individual menstrual cycle recordings collected to date revealed intermittent synchronicity between the lunar and menstrual cycles (Helfrich-Förster et al., 2021). Menses onset and offset were recorded for up to 32 years (15 years on average) in 22 healthy German women, free from hormonal contraceptives, aged 14 to 45 years. This longitudinal data set showed that while menstrual cycles varied in length within individuals, women who had menstrual cycles longer than 27 days were intermittently synchronized to the lunar light and gravimetric cycles (Helfrich-Förster et al., 2021). This was most evident in younger women (<35 years), whose menstrual cycles synchronized with the new or full moon (predominantly the full moon phase) ~23% of the recorded time, on average. These findings support the idea that lunar and menstrual cycles are related. Still, replication studies in larger samples sizes are required to further examine the relationship between moonlight and human biological rhythms.

### The Relationship Between Sunlight and the Menstrual Cycle

The relationship between sunlight and human biological rhythms has also been explored, notably in studies assessing the relationship between photoperiod (duration of available sunlight) and menstrual cycles. Photoperiod lengths differ across the seasons, with longer photoperiods observed in spring and summer and shorter photoperiods observed in autumn and winter. Vitamin D, also known as the sunshine vitamin (Wacker and Holick, 2013), has been shown to play a role in female reproduction, with vitamin D supplements aiding menstrual cycle regularity and fertility (Lerchbaum and Obermayer-Pietsch, 2012). Lower vitamin D levels in women have also been associated with longer follicular phases and longer menstrual cycle lengths (Jukic et al., 2018). Irregular menstrual cycle lengths and those that are shorter or longer than normal (typically defined as ~21-35 days with <9 days variance between cycles) are associated with higher infertility and premature mortality rates (Bradley et al., 2021; Kolstad et al., 1999; Wang et al., 2020). The effects of sunshine were studied in 129 healthy Russian women aged 18 to 40 years, free from hormonal contraceptives and reporting regular menstrual cycle lengths of 21 to 36 days (Danilenko et al., 2011). Ultrasounds were used to confirm the absence of reproductive issues in the study participants. Measurements of FSH and ovarian follicles were collected during two time points (summer and winter) across the year (Danilenko et al., 2011). Results showed that FSH levels and ovarian follicle size increased during the summer, ovulation occurred more frequently, and menstrual cycles were shorter compared with winter. Importantly, moon phase and moonlight were not significant predictors in this cohort (Danilenko et al., 2011).

The relationship between sunlight and human biological rhythms is ancient. The earliest known record of the relationship between photoperiod and the menstrual cycle comes from Frederick Cook’s anecdotal observations during his Arctic expedition in 1897. Cook claimed that Inuit women did not menstruate during the 4 months of darkness that blankets the skies of the northwest coast of Greenland. When the sunlight returns in spring, Inuit women begin to menstruate again, and children are typically born 9 months later (Cook, 1897). Importantly, Cook claimed to observe similar effects among his own team, with sexual desire and menstruation decreasing in the winter months, when sunlight was absent. Although Cook did not collect or present any data in these women, this anecdotal observation suggests that the availability of sunlight (photoperiod) affects neuroendocrine function (Cook, 1897; Llewellyn, 1932).

This early anecdotal observation has since been confirmed in several studies using more controlled measures. In a group of 32 naturally cycling Finnish women, blood samples were collected every 2 h across 24 h at two time points: the dark season (November-January) and the light season (February-October). In addition, urine samples were collected twice a day over 24 h during both dark and light seasons, and each woman was studied during the same menstrual phase in each season, typically during the luteal phase. Samples were assayed for FSH, LH, estradiol, progesterone, and testosterone using radioimmunoassay (RIA; Kauppila et al., 1987). The researchers discovered that during the dark season, melatonin concentration increased, and ovarian function decreased in the women. Conversely, melatonin concentration decreased, and ovarian function increased during the light season, demonstrating the influence of seasonal light exposure on the menstrual cycle. These findings were replicated in a smaller sample of naturally cycling Finnish women (*n* = 12; Kivelä et al., 1988). Blood samples were again collected over 24 h across the dark and light seasons, but the sampling rate was 6 h instead of 2 h. Sampling occurred on days 10, 14, and 25 of the menstrual cycle in both seasons and assayed for melatonin, prolactin, FSH, and LH using RIA. Melatonin and LH concentration was shown to be higher in winter compared with summer, but there were no effects of the seasons on FSH or prolactin concentration (Kivelä et al., 1988). More recently, it has been shown that FSH increases, ovulation rates increase, and menstrual cycles are shorter in summer compared with winter (Danilenko et al., 2011). These findings suggest a relationship between the length of the photoperiod and the menstrual cycle in women.

Findings from non-human animal research, predominantly studies in rodents, show that there is a relationship between melatonin and sex-steroid hormones, suggesting that melatonin plays a role in sexual maturation and reproduction (Gauer et al., 1994; Heideman and Bronson, 1990; Ishizuka et al., 2000; Karatsoreos et al., 2007; Weaver et al., 1989). The work here is rich and as such, beyond the scope of this review, but it is well worth mentioning that a key insight gained from the non-human animal research is that melatonin seems to have antigonadotropic effects, reducing ovulation (Blask and Nodelman, 1979; Brzezinski et al., 1987; Dair et al., 2008; Reiter et al., 1979; Richardson et al., 1981; Soares et al., 2003; Tamura et al., 2009), which neatly aligns with the findings that increased melatonin secretion during dark seasons decrease ovarian function in women (Kauppila et al., 1987; Kivelä et al., 1988).

The relationship between melatonin and sex-steroid hormones in humans has been explored to a lesser degree than in non-human animals, but the findings are comparable. Notably, melatonin levels in humans are generally highest during childhood and gradually decline in both sexes during the onset of puberty (Cipolla-Neto et al., 2022; Crowley et al., 2012; Luboshitzky and Lavie, 1999; Reiter, 1998) until old age (Brzezinski, 1997; Scholtens et al., 2016; Waldhauser et al., 1988), suggesting that melatonin plays a role in sexual maturation and reproduction in humans as well. Administration of daily exogenous melatonin (300 mg) in 12 healthy young women (aged 18-37 years) with regular menstrual cycles (ranging 22-34 days), free from hormonal contraceptives across 4 months showed that LH, estradiol, and progesterone levels were significantly reduced by the fourth month compared with the first month (Voordouw et al., 1992). These results were partially confirmed in another randomized clinical trial that showed administration of lower doses of exogenous melatonin (3 mg) to perimenopausal women (aged 43-49 years) over a 6-month period reduced LH levels (Bellipanni et al., 2001).

Women with polycystic ovarian syndrome (PCOS), a complex neuroendocrine disorder affecting up to 20% of women during reproductive age, typically experience irregular menstrual cycles and other fertility problems, hyperandrogenism (Mojaverrostami et al., 2019), and an increased risk of mood disorders (e.g., depression and anxiety; Damone et al., 2019; Deeks et al., 2010; Kerchner et al., 2009), metabolic disorders (e.g., obesity and Type II diabetes; De Groot et al., 2011; Naderpoor et al., 2014), and sex-specific cancers (e.g., endometrial cancer; Dumesic and Lobo, 2013; Meczekalski et al., 2020). Generally, women with PCOS have altered melatonin levels compared with women without PCOS, with reports of higher serum and urinary melatonin levels (Jain et al., 2013; Luboshitzky et al., 2001; Mojaverrostami et al., 2019; Shreeve et al., 2013) and lower melatonin levels in follicular fluid (Mojaverrostami et al., 2019; Tamura et al., 2009). The exact nature of melatonin and its role in PCOS is still being explored, but there is some good evidence that melatonin could be used to treat menstrual cycle irregularities, hyperandrogenism, and fertility issues (Jamilian et al., 2019; Kim et al., 2013; Tagliaferri et al., 2018), further cementing its role in sexual maturation and reproduction.

There is compelling evidence that melatonin is an antiestrogen and inversely related to estrogen from studies assessing breast cancer in women (Cipolla-Neto et al., 2022; Sánchez-Barceló et al., 2005). Estradiol has been shown to play a role in the development of breast tumors (Cauley et al., 1999; Hayashi et al., 2003; Sasano et al., 2006; Yue et al., 2010), whereas melatonin has been shown to have anticarcinogenic properties (Karbownik et al., 2001; Srinivasan et al., 2008), reducing the levels of estradiol. Studies in totally blind women illustrate that they are at a lower risk of breast cancer and seem to be protected from its development (Coleman and Reiter, 1992; Feychting et al., 1998; Flynn-Evans et al., 2009), presumably because blind people have more melatonin circulating through their body than sighted people (i.e., less suppression of melatonin in blind women). There is also some evidence that women working night shifts (e.g., nurses) who are exposed to light at night on a regular basis might be at a greater risk of developing various cancers (Davis et al., 2001; Schernhammer et al., 2013), and although further work in this area is required, shift work has been classified as probably carcinogenic by the International Agency for Research on Cancer (Erren et al., 2019).

### The Relationship Between Electric Light and the Menstrual Cycle

In addition to these studies assessing the relationship between natural light and the menstrual cycle, several studies have assessed the relationship of electric light at night and the menstrual cycle. A case study in a 26-year-old woman with an irregularly long menstrual cycle ranging 33 to 48 days showed that when this individual was exposed to 235 lux throughout the night on days 14 to 17 of their menstrual cycle, their cycle length was reduced to 29 to 35 days (Dewan, 1967). Light was administered via a common bedroom lamp placed on the floor at the foot of the participant’s bed. The bedroom lamp had a shade that projected light to the ceiling and walls, using an incandescent bulb. The authors do not state the rationale for using 235 lux, nor is there any explanation as to how ovulation was confirmed after exposure to electric light at night. Dewan and colleagues (1978) followed up on this study in 15 new women who reported irregularly long menstrual cycles. Using 29 days as normal cycle length, light exposure of 235 lux was administered via a table lamp fitted with an incandescent bulb, placed on the floor about 10 feet from the participants’ bedhead on nights 14 to 17 to induce ovulation. The majority of women (9 out of 11; ~82%) exposed to light at night had shorter menstrual cycles, confirming the novel findings from the earlier case study (Dewan et al., 1978). The authors seemed to confirm ovulation using basal body temperature recordings and suggest that future studies should use more reliable measures of ovulation, such as serum LH via RIA.

Dewan’s work has since been replicated by other researchers. Using a more controlled experimental design, Lin and colleagues assessed the effects of electric light at night in 16 American women aged 18 to 30 years with long or irregular menstrual cycles ranging 33 to 48 days. Women in the study were required to keep track of their menstrual cycles for three consecutive cycles prior to receiving one of two experimental light treatments. Once menstrual records were collected, the women were randomly assigned to either the active treatment (*n* = 7) or placebo treatment (*n* = 9). Participants in the active treatment were exposed to 235 lux administered via a bedside lamp (placed 1 m away from the participant), which was fitted with a white incandescent bulb during nights 13 to 17 of their menstrual cycles. Those in the placebo treatment were exposed to significantly dimmer light of 1.7 lux administered via bedside lamp (placed 1 m away from the participant), which was fitted with a red photographic safe light during nights 13 to 17 of their menstrual cycles (Lin et al., 1990). The women in the active treatment showed significantly shorter cycles compared with those in the placebo treatment, lending support to the notion that light exposure at night prior to ovulation shortens the menstrual cycle in women (Lin et al., 1990). Subsequent studies provide evidence of light exposure at night effectively shortening the menstrual cycles in women with irregularly long cycles, suggesting that electric light can indeed be used as an effective treatment for menstrual cycle irregularities (Putilov et al., 2002; Rex et al., 1997). A limitation of these earlier studies is that we cannot know the m-EDI for studies where only photopic lux is provided (Table 1). This highlights the need for studies using light as an intervention to properly document the properties of the light source used (Spitschan, 2019b; Spitschan et al., 2019).

**Table 1.** Summary of key studies assessing the relationship between light and the menstrual cycle.

Study Details	Study Type	Light Source	Participants	Methods	Findings
Law (1986), China	Observational	Moonlight	826 females aged 16-25 years; normal menstrual cycles (defined as 26-35 days)	Experiment 1: menstrual data reported over four lunar months across various seasons in 1982	Close to one-third of women in this sample menstruated at the new moon
Cutler et al. (1987), United States	Observational	Moonlight	838 females aged ~18-26 years; naturally cycling with data from those exhibiting a 29.5 ± 1 day cycle (~27% of total sample) analyzed	Menstrual and sexual events recorded over ~14 weeks during the fall and spring in 1976, 1977, 1979 and 1983	69% of the selected sample (*n* = 229) menstruated within 7.5 days of the full moon
Ilias et al. (2013), Greece	Observational	Moonlight	74 females aged from pool with age range 14-29 years; normal menstrual cycles (defined as 21-35 days)	Menstrual data reported over 1 year	No evidence of a correlation between menstruation and moon phase
Science Writers at Clue (2016), International	Observational *(non-peer reviewed)*	Moonlight	1.5 million females presumably of reproductive age (not defined); naturally cycling	Menstrual data reported over 7.5 million cycles using Clue smartphone app	No evidence of a correlation between menstruation and moon phase
Komada et al. (2021), Japan	Observational	Moonlight	529 females aged 25-39 years; normal menstrual cycles (defined as 25-38 days)	Menstrual data reported over 6 months using Luna Luna smartphone app	Moon phase did not influence the onset of menstruation
Helfrich-Förster et al. (2021), Germany	Longitudinal	Moonlight	22 females aged 14-45 years; naturally cycling	Menstrual data reported for up to 32 years (average of 15 years)	Women with menstrual cycles > 27 days, especially those aged <35 years, intermittently synchronized with the new and full moon (predominantly the latter phase)
Kauppila et al. (1987), Finland	Observational	Sunlight	32 females aged 22-42 years; naturally cycling	Measures of FSH, LH, estradiol, progesterone, testosterone, and melatonin were taken in autumn/winter (dark season) and spring/summer (light season)	Ovarian function decreased and melatonin concentration increased during the dark season. During the light season, ovarian function increased, and melatonin concentration decreased
Kivelä et al. (1988), Finland	Observational	Sunlight	12 females aged 23-35 years; naturally cycling	Measures of FSH, LH, prolactin and melatonin were taken in autumn/winter (dark season) and spring/summer (light season)	Melatonin and LH concentration was higher in winter compared with summer. No effect of the seasons on FSH or prolactin concentration
Danilenko et al. (2011), Russia	Observational	Sunlight	129 females aged 18-40 years; naturally cycling; normal menstrual cycles (defined as 21-36 days)	Measures of FSH, ovarian follicle size, and ovulation were taken in winter and summer	FSH and ovarian follicle size increased, ovulation occurred more frequently, and menstrual cycles were shorter by nearly 1 day on average in the summer compared with the winter
Dewan (1967), United States	Case study	Electric light at night	One female aged 26 years; irregular menstrual cycle (defined as 33-48 days)	Exposure to 235 lux during nights 14-17 of menstrual cycle (around the time of ovulation in a normal 29 day cycle)	Light exposure reduced menstrual cycle length to normal range (29-35 days)
Dewan et al. (1978), United States	Intervention	Electric light at night	15 females aged 21-36 years; irregular menstrual cycle (defined as 33-48 days)	Exposed to 235 lux during nights 14-17 of menstrual cycle	Light exposure reduced menstrual cycle length to normal range (29-35 days) in most women
Lin et al. (1990), United States	Intervention	Electric light at night	16 females aged 18-30 years; irregular menstrual cycle (defined as 33-48 days)	Menstrual data collected for three consecutive months. Randomly allocated to active treatment (exposure to 235 lux; *n* = 7) and placebo treatment (exposure to 1.7 lux) on nights 13-17 of menstrual cycle	Women treated with 235 lux had shorter menstrual cycles, on average (~13 days) than women in the placebo group.
Rex et al. (1997), United States	Intervention	Electric light at night	18 females aged 18-35 years; irregular menstrual cycle (defined as 33-60 days); free from hormonal contraceptives for at least 6 months	Menstrual data collected over four consecutive cycles. Randomly allocated to 235-250 lux white light on nights 13-17 of one menstrual cycle and < 1 lux red light on nights 13-17 of another menstrual cycle (counterbalanced)	Exposure to 235-250 lux significantly reduced menstrual cycle length. Exposure to <1 lux red light also shortened menstrual cycle length compared with baseline
Putilov et al. (2002), Russia	Intervention	Electric light at night	25 females aged ~25 years on average; irregular menstrual cycle (defined as 36-53 days); naturally cycling	Light exposure of 240 lux for 5 consecutive nights during early, middle, and late phase of menstrual cycle. Wash out period of at least two untreated cycles before and after light exposure at each phase	Exposure to 240 lux significantly reduced menstrual cycle length. No significant difference in effects of light between early, middle, and late phases of menstrual cycle

## Is there a sex difference in the physiological effects of light?

The short answer to this question is that we do not know. An early study assessing the effects of light on melatonin production exposed 10 healthy Australian women (*n* = 5) and men (*n* = 5) with an average age of 32 years to light intensities of 1000, 1500, 2000, and 2500 lux administered via a tungsten filament lamp with dimmer capabilities to alter intensities. Light exposure commenced at midnight and concluded at 0200 h for each participant, with a 5-day interval between the four light conditions. Blood samples were collected during each light exposure and assayed for melatonin. Melatonin levels between women and men were assessed using contrast analysis and showed no difference between the sexes (Boyce and Kennaway, 1987). Another study in 13 healthy Australian women (*n* = 4) and men (*n* = 9) assessing the effects of moderate and bright light (200-3000 lux via eight fluorescent tubes) on melatonin suppression did not analyze their data by sex, presumably due to the small sample size (McIntyre et al., 1989). To date, only a few studies have specifically examined sex differences in the response of the circadian system to light, and the ones that have did not account for menstrual phase (Nathan et al., 1997) or tested women in the follicular phase only (Monteleone et al., 1995). An early study in a small, randomized sample of 12 healthy, young Italian women (*n* = 6) and men (*n* = 6) aged 22 to 34 years, demonstrated greater circadian sensitivity in women to bright light (2000 lux via two full spectrum fluorescent lamps) than men. A difference of 40% more melatonin suppression in women was reported by the authors (Monteleone et al., 1995). Others found no sex differences in light sensitivity, measured using melatonin suppression at moderate to bright light (Nathan et al., 1997).

The conflicting findings from these studies may be due to methodological discrepancies in the experimental protocols. Menstrual phase, for example, was not accounted for in the study reporting no sex differences in melatonin suppression and light sensitivity (Nathan et al., 1997). In addition, the researchers of the study that reported no sex differences had a broad age range (20-56 years in experiment 1; 25-51 years in experiment 2), and as such, included premenopausal, perimenopausal, and menopausal women in their analyses (Nathan et al., 1997). Any potentially observable sex differences in this study may have been diluted or masked due to the variable range in hormones within and between women. Likely, comparing the results of women of reproductive age with those of women in the perimenopausal and menopausal stages obscured their results.

The study that did report greater light sensitivity in women only assessed women in the follicular phase of the menstrual cycle but did not report how menstrual phase was determined (Monteleone et al., 1995). This discrepancy makes it difficult to compare and interpret the findings between the studies. One of the key problems is that the thresholds used to determine normal estradiol and progesterone levels are not explicitly stated, and different laboratories use different thresholds (Allen et al., 2016). The determination of menstrual phase is also ambiguous in these studies. Here too, the authors of these studies provide little information about the properties of the light source, making it difficult to determine the m-EDI. Small sample sizes, which are a common feature in human studies of light, melatonin, and the menstrual cycle limit the generalizability of these findings.

## Does the menstrual cycle affect or modulate the physiological effects of light?

We are aware of one study that has directly assessed whether the menstrual cycle affects the circadian system’s response to light in humans. A study conducted by Nathan and colleagues (1999) specifically examined the role of the menstrual cycle on light sensitivity in six healthy Australian women aged 18 to 35 years, free from hormonal contraceptives (Nathan et al., 1999). Each woman was tested during menses, the follicular phase, and the luteal phase of their cycles. During each test session, women were exposed to 200 lux (administered via two 500 Kelvin fluorescent tubes) for 1 h between midnight and 0100 h, with hourly blood samples collected before, during, and after the 1-h light exposure. In this study, melatonin suppression, which was used to determine light sensitivity, was not significantly different at each phase of the menstrual cycle tested (Nathan et al., 1999).

## The File Drawer Problem

It is likely that other researchers have looked at sex differences and the effects of menstrual phase in light sensitivity and, finding no difference, did not publish their null results due to publication bias. Researchers often refrain from writing and submitting null findings, as the likelihood of publication is notably less than reports of significant and novel findings (Easterbrook et al., 1991; Franco et al., 2014; Rosenthal, 1979), especially in psychology, with earlier reports showing that of 692 papers that were published in four journals, ~92% rejected the null hypothesis (Hubbard and Armstrong, 1992, 1997).

A prominent debate in chronobiology regarding the file drawer problem was raised when two separate groups of researchers analyzed their sleep data retrospectively to assess the effect of the lunar cycle on sleep in humans. In 2013, Cajochen and colleagues retrospectively analyzed sleep data collected during a constant routine protocol, spanning 3.5 days (Cajochen et al., 2013). Data from 17 healthy young adults (18-31 years; women = 9, men = 8) and 16 healthy older adults (57-74 years; women = 8, men = 8) showed that during the full moon, SWS decreased by 30%, sleep onset latency increased by 5 min, and total sleep duration decreased by 20 min (Cajochen et al., 2013). Encouraged by these results, Cordi and colleagues (2014) retrospectively analyzed sleep data in a much larger sample of participants (*n* = 1265) across three experiments. The researchers did not find any effect of the lunar cycle on the sleep parameters (duration of SWS, sleep onset latency, total sleep duration) reported by Cajochen and colleagues (Cordi et al., 2014).

The conflicting findings can be partly attributed to methodological discrepancies. Cajochen and colleagues (2013) utilized a constant routine protocol, which is a highly controlled experimental design used to assess endogenous rhythms by keeping environmental cues such as ambient light, room temperature, meal timing, caloric intake, posture, and activity stable (Blatter and Cajochen, 2007; Minors and Waterhouse, 1984). Participants in the studies by Cordi and colleagues, by contrast, arrived at the sleep laboratory a few hours prior to habitual bedtime in the first two experiments (*n* = 395), while the third experiment used portable sleep monitors, allowing participants to sleep at home (*n* = 870; Cordi et al., 2014). The use of portable sleep monitors in collecting most of their sleep data is not ideal, as there is lower experimental control, making it difficult for researchers to monitor and confirm compliance to the experimental protocol, a point conceded by the research team (Cordi et al., 2014). Differences in the duration of the respective studies, and sleep and circadian measures also contribute to the conflicting findings (Cajochen et al., 2013, 2014; Cordi et al., 2014). More recently, a detailed field study among the indigenous Toba/Qom of Argentina showed that individuals had later bedtimes and slept less in the days leading to a full moon, when there is more natural light at night (Casiraghi et al., 2021). Those individuals who had no access to electric light experienced the most significant changes to their sleep patterns, providing evidence that lunar cycles affect sleep in humans. The availability of moonlight seems to increase activity, resulting in later bedtimes and shorter sleep duration (Cajochen et al., 2013; Casiraghi et al., 2021).

Despite the conflicting findings, both research groups provided detailed reports of their studies, allowing easier comparisons for review. Furthermore, if Cajochen and colleagues had not published their findings, Cordi and colleagues’ impressively large data set would have remained locked away in their file drawer.

We must publish null results and re-examine data sets when new information is brought to light. One strategy for ensuring that data sets are not locked away in file drawers is to make data publicly accessible by routine. Researchers often have a specific goal in mind when conducting experiments. Human research is painstaking and time-consuming. Open-access data allow researchers to make their data sets available to others who are interested in exploring various questions outside the scope of what was originally published. It can also aid researchers who might not have the means of collecting their own data and create a more collaborative community globally.

## Discussion and Future Research

In this article, we showed that there is evidence of an association between light and the menstrual cycle, but the nature of this relationship is dependent on the timing of the light (e.g., sunlight exposure during the day vs. electric light exposure during the night). The most compelling evidence consistently shows that longer photoperiods increase ovarian function, while shorter photoperiods decrease ovarian function (Danilenko et al., 2011; Kauppila et al., 1987; Kivelä et al., 1988; Llewellyn, 1932). The studies assessing the relationship between the amount of sunlight present in the natural environment (photoperiod) and reproductive function are the most controlled, with precise measures such as ultrasounds and biological samples for sex hormone assays used in conjunction with self-reports. There is modest evidence that electric light exposure at night can be used to reduce the menstrual cycle in women experiencing irregularly long menstrual cycles, but these studies are not as controlled, often lacking objective measures of menstrual phase and wholly relying on self-reports. Determination of menstrual phase is not clear and study procedures are not well-defined in these studies, which makes it difficult to understand, interpret, and compare findings. At this point in time, evidence of a relationship between lunar cycles and menstrual cycles is inconclusive. The earliest experiments provided little information on who their participants were, how menstrual cycles were determined, what constituted an irregular menstrual cycle, and the rationale for omitting two-thirds of their data to confirm their hypothesis (Cutler et al., 1987). Currently, the relationship between the lunar cycle and menstrual cycle is still an area that needs further exploration.

The effect of the menstrual cycle on light sensitivity is also an area that is lacking in evidence. Few studies have specifically assessed sex differences in the human circadian system’s response to light, and one of these studies has assessed light sensitivity across the menstrual cycle (Nathan et al., 1999). There are conflicting findings regarding sex differences in light sensitivity, with the majority of studies reporting no sex differences (Boyce and Kennaway, 1987; Nathan et al., 1997) and one reporting greater light sensitivity in women during the follicular phase (Monteleone et al., 1995). Menstrual phase was shown to have no effect on melatonin concentration in a small sample (*n* = 6) of premenopausal women, free from hormonal contraceptives (Nathan et al., 1999). While this study provides some details on how menstrual phase was determined (biological samples collected for progesterone assays), it is not clear if participants were keeping track of their menstrual cycles prior to enrolment in the study and during the study. Furthermore, the study design has some flaws. To begin, light exposure was short, lasting a single hour and timed at midnight for all women, irrespective of habitual bedtime. The authors’ rationale for this method of delivering the experimental light assumes that melatonin suppression is greatest during the middle of the biological night for all individuals. A more accurate measure would be to time the light exposure based on the individual’s habitual bedtime. This could be achieved by asking participants to keep to a regular sleep-wake schedule of their choosing for at least 1 week prior to the experimental light exposure. Bed and wake times can be recorded daily using sleep diaries and compliance to the sleep-wake schedule can be confirmed via actigraphy. Menstrual phase can also be recorded on these sleep diaries, with participants recording the onset and offset of menses.

Studies demonstrating that a moderate light intensity of 235 lux can shorten the menstrual cycle in women with irregularly long menstrual cycles are important as they show that light can be used as a form of treatment (Dewan, 1967; Dewan et al., 1978; Lin et al., 1990). It is not clear, however, whether this light treatment subsequently affects sleep parameters in women. If a woman were exposed to 235 lux prior to bedtime, it may indeed delay sleep onset and shorten their sleep period. Future research would benefit from exploring the effect of lower light intensities (<100 lux) on menstrual cycle length so that light treatment for menstrual irregularities can be provided without negatively affecting sleep.

Light sensitivity may be influenced by biological sex and menstrual phase but there might not be any effect at all. To move the needle forward on this topic, we must incorporate certain measures so that we avoid repeating mistakes of past research studies. Researchers often leave readers guessing the methods used to determine menstrual phase, which leads to misinterpretation and confusion. Collecting data about reproductive health and possible issues can be sensitive for both researchers and participants, with studies showing an effect of experimenter sex on participant responses in scientific studies (Chapman et al., 2018; Dolgun et al., 2014; Gott et al., 2004; Mengesha et al., 2018; Vigil et al., 2015). Female participants might be reluctant to enroll in studies led by male researchers if they feel uncomfortable, but with increasing numbers of female researchers and more open public discourse on the topic of menstruation (e.g., campaigns designed to remove the stigma around menstruation), this problem may soon dissipate.

Any researcher enrolling female participants must collect and report basic information about their age, menses onset and offset, use of hormonal contraceptives, use of menopausal hormonal therapy (hormone replacement therapy), and other sex steroid use. Collecting these data provides basic descriptives of the cohort being studied. The experimenter could ask about these descriptives during screening (e.g., phone interview or in-person) and treat them as covariates in their analyses (e.g., differences between sexes or between women). At present, there are no clear cut-offs for the determination of the reproductive states in women, but providing a range of ages, for example, in publications will help other researchers use similar cut-offs in their own studies and a consensus can be achieved over time. When studying cisgender women and non-binary individuals born female who menstruate, researchers should at the very least report the measures used to determine menstrual phase (e.g., self-reports, biological samples, LH kits), average cycle lengths, and how the menstrual phases were defined. Even if the definition of menses onset/offset are unclear now, simply reporting how menses was determined will help other researchers interpret the methods, which can ultimately help establish uniformity between laboratories. At present, the key task for researchers is to collect and provide as much information as is relevant to the research question. Researchers interested in assessing menstrual phase in naturally cycling women should refer to the detailed and engaging review provided by Allen and colleagues (2016).

Describing participants in some detail will allow readers to better understand the results of the studies. For example, if researchers publish the age range and mean of their female participants, the reader can determine whether the participants are premenopausal or postmenopausal. A simple statement about the presence or absence of hormonal contraceptive use will allow the reader to determine whether the results were affected by exogenous sex steroids. Describing participants in terms of biological sex assigned at birth and gender or non-binary identification can also be meaningful when interpreting results, allowing researchers to assess how societal roles or the use of exogenous sex steroids influence sleep and circadian rhythms in women. Researchers can be more thoughtful in the conception, design, and presentation of their experiments by becoming aware of the nuances within and between humans, which will benefit biomedical research at large.

It is also important that all researchers, irrespective of their own sex and gender identity, understand the unique features of the menstrual cycle and how it can potentially affect study outcomes. In addition to the topic of menstrual cycles, further investigations regarding the relationship between the circadian system and menopause, pregnancy, and the use of hormonal contraceptives will allow us to better understand individual differences in light sensitivity. Researchers can no longer exclude women from participation in scientific experiments, as both funding and publication agencies require the inclusion of all sexes and encourage analysis of sex. When creating experimental protocols, researchers must carefully consider their research question and study outcomes, collect the relevant data, and report their findings with greater transparency, even if they cannot entirely make sense of them (somebody else reading their work might have some insights). For an eloquent and comprehensive review of sex and gender differences in biomedical research and thoughtful experimental designs, we recommend referring to Rich-Edwards and colleagues (2018).

When it comes to understanding the underlying mechanisms of individual differences in light sensitivity, we are still in the early stages of discovery. Therefore, it is vital that chronobiologists avoid hastily providing recommendations and guidelines for architectural lighting design industries and instead step back and carefully examine and understand the evidence at hand.
